# Active Compounds, Targets, and Mechanisms of *Salvia miltiorrhiza* Bunge in Treating Interstitial Cystitis/Bladder Pain Syndrome

**DOI:** 10.1002/iid3.70173

**Published:** 2025-04-14

**Authors:** Liang Wang, Bei Yu, YaRong Wang, Xi Qu, Wei Tang

**Affiliations:** ^1^ Department of Urology The First Affiliated Hospital of Chongqing Medical University Chongqing China

**Keywords:** interstitial cystitis/bladder pain syndrome, luteolin, network pharmacology, PI3K‐Akt signaling pathways, *Salvia miltiorrhiza* Bunge, target

## Abstract

**Objective:**

To investigate the active compounds, molecular targets, and biological mechanisms of *Salvia miltiorrhiza* Bunge (SM) in treating interstitial cystitis/bladder pain syndrome (IC/BPS) through network pharmacology and a cyclophosphamide‐induced cystitis model.

**Methods:**

A network pharmacology approach was used to assess the effects of SM and luteolin in IC/BPS. Female C57BL/6 mice were divided into four groups: CON, CON + Luteolin, CYP, and CYP + Luteolin, with luteolin (100 mg/kg) administered for CYP‐induced cystitis. Histological and molecular analyses, including H&E staining, TUNEL, ELISA, Western blot, and urodynamics, were performed to explore the mechanisms.

**Results:**

Network pharmacology showed 65 active ingredients and 148 potential targets of SM in the treatment of IC/BPS, of which luteolin had the highest potential. TP53, AKT1, CCND1, EGFR, and ERBB2 are the core targets, and PI3K‐Akt and p53 are important signaling pathways for luteolin in the treatment of IC/BPS. Compared with the CYP group, the CYP + Luteolin group showed significantly lower bladder tissue scores; reduced expression of malondialdehyde, inflammatory factors (IL‐18, IL‐1β, IL‐6), and apoptosis‐related proteins (cleaved‐Caspase‐3, Bax, cleaved‐Caspase‐8); significantly increased expression of total SOD and glutathione; and improved bladder function. Animal experiments have shown that luteolin can block the activation of the PI3K‐Akt and p53 signaling pathways.

**Conclusion:**

SM has a variety of potentially active components for the treatment of IC/BPS, of which luteolin has the highest potential. Luteolin can inhibit inflammation, oxidative stress, and apoptosis through the p53 and PI3K‐Akt signaling pathways and plays a role in treating IC/PBS.

## Graphical abstract

*Salvia miltiorrhiza* can play the role of treating interstitial cystitis/bladder pain syndrome through multiple targets and multiple components. Luteolin is the most potential active ingredient of *S. miltiorrhiza* in the treatment of interstitial cystitis/bladder pain syndrome. Luteolin can inhibit oxidative stress, inflammation and apoptosis through PI3K Akt and p53 signaling pathways to treat interstitial cystitis/bladder pain syndrome.

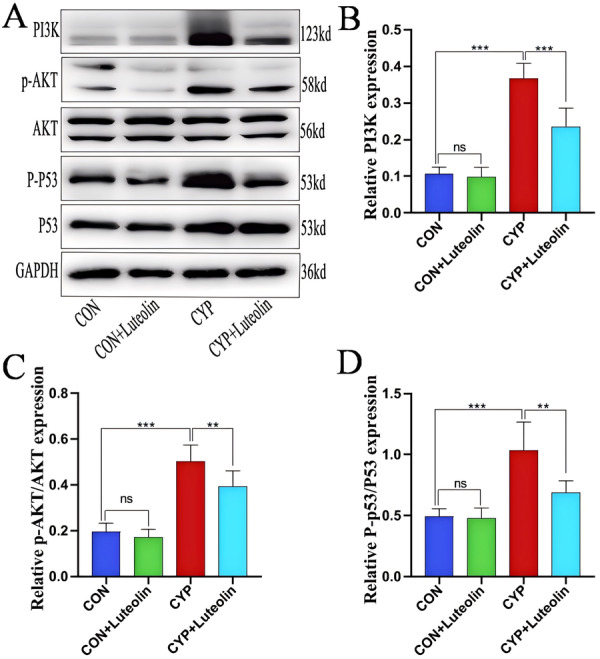

## 1 Introduction

Interstitial cystitis/painful bladder syndrome (IC/BPS), a clinical syndrome with an unknown cause, is characterized by symptoms such as urinary urgency, urinary frequency, and suprapubic pain that intensify with bladder filling and are alleviated by urination [1]. The incidence of IC/PBS is related to race, age, and sex. The incidence among American women is 2.7%–6.5%, and the majority of them are aged 30–50, with White women accounting for more than 90% [2]. The incidence in females is 3–4/100,000 in Japan and 18/100,000 in Europe, and is five times that in males [1, 2, 3]. The occurrence of IC/PBS significantly impacts the psychological well‐being and quality of life of patients, with the rate of pain‐induced suicide being four times higher than that of healthy individuals [4, 5]. Currently, the main therapies for IC/PBS include oral drugs, bladder instillation, bladder ulcer resection, and sacral neuromodulation. These therapies are mainly used to relieve the clinical symptoms of the patients [4, 5]. Owing to the unclear pathogenesis and lack of specific pathophysiological changes in IC/PBS patients, no complete cure is currently available for IC/PBS [6, 7]. Thus, understanding the underlying mechanisms of IC/BPS and discovering novel therapeutic options is of paramount clinical importance.

Chinese herbal medicines have been used in China for thousands of years, and the development and research of natural active ingredients in Chinese herbal medicines can provide new ideas and treatment options for the intervention and treatment of IC/PBS. Network pharmacology is an innovative approach that elucidates drug treatment mechanisms by constructing “drug‐target‐disease” networks and analyzing network topologies [8]. This holistic method has significantly advanced the study of active components, targets, and mechanisms in Chinese herbal medicines [8, 9, 10], notably *Salvia miltiorrhiza* Bunge (SM), which is widely used to treat conditions such as cardiovascular diseases, diabetes, arthritis, and asthma [8, 9, 10, 11]. Studies have shown that the active ingredients in SM, such as tanshinone, salvianol, and luteolin, have anti‐inflammatory, antioxidant, anti‐fibrotic, and cardiovascular protective effects [9, 10, 11, 12, 13]. However, whether SM has potential therapeutic value in IC/PBS has not been reported. Accordingly, this study used a network pharmacology approach to explore the active components and core targets of SM in the treatment of IC/BPS. At the same time, the authors further verified the core components, targets, and molecular mechanisms using animal models, laying a theoretical foundation for the application of SM in the treatment of IC/PBS.

## 2 Methods

### 2.1 Network Pharmacology Analysis of SM

The chemical constituents of SM were identified using the Traditional Chinese Medicine System Pharmacology Database and Analysis Platform. We screened for compounds with an oral bioavailability (OB) of at least 30% and drug‐like properties (DL) of at least 0.18, resulting in 65 potential active ingredients and their corresponding target proteins. Further refinement was conducted using the UniProt database to verify and correct the target protein and gene information for these main components.

### 2.2 IC/BPS Target Screening and Intersection Target Protein–Protein Interaction (PPI) Network Construction

GeneCards, OMIM, TTD, DrugBank, and DisGeNET databases were searched to collect IC/BPS targets, with “Bladder Pain Syndrome” and “Interstitial Cystitis” as the keywords. Targets with a score > the median in the GeneCards database were selected as potential targets for IC/BPS. The disease database targets were merged, gene information was corrected in the UniProt database, and the potential targets of IC/BPS were identified after searching and proofreading. The common predicted targets of IC/BPS and SM were obtained using the intersection of the mapping tool of the Venny 2.1 online software, and the “drug‐component‐target‐disease” network diagram was constructed using the Cytoscape 3.8.2 software. A network Analyzer was used to perform topological analysis on the network graph to identify the core components of SM in the treatment of the interstitial bladder. Through topological analysis, the authors found that luteolin was the core active ingredient in the treatment of IC/BPS, then analyzed the common predicted targets of luteolin and IC/BPS, and thereby constructed the “disease‐target‐component” network diagram and protein‐interaction network diagram of luteolin. Finally, a topology analysis was performed using the Network Analyzer tool. Genes that exhibited a degree value above the average were designated as core targets following degree sorting. A bar graph visualizing these targets was generated using R version 4.0.5.

### 2.3 Gene Ontology (GO) and Kyoto Encyclopedia of Genes and Genomes (KEGG) Pathway Enrichment Analysis

The common targets between the drug and disease were subjected to functional enrichment analysis, including biological process (BP), molecular function (MF), cellular component (CC), and KEGG pathway enrichment using the GO framework. Using R software and the Bioconductor package, we performed GO and KEGG enrichment analyses on key target genes, setting significance thresholds at *p* < 0.05 and *Q* < 0.05. The results were visualized as bar graphs to clearly depict the findings.

### 2.4 Establishment of an Animal Model of CYP‐Induced Cystitis and Treatment With Luteolin

Female C57BL/6 mice, aged 10–12 weeks, were procured from the First Affiliated Hospital of Chongqing Medical University. This animal research received ethical clearance from the Laboratory Animal Welfare and Ethics Committee of the same institution (Approval CYFY‐20230109), ensuring compliance with Animal Welfare Guidelines and the Declaration of Helsinki. As reported in prior studies [14, 15], cyclophosphamide (CYP; 150 mg/kg, Sigma‐Aldrich, St. Louis, MO, USA) was administered to induce cystitis. The study involved 72 mice, which were systematically allocated into four groups: CON (control), CON + luteolin, CYP, and CYP + luteolin, each consisting of 18 mice. For a period of 7 days, the luteolin‐treated groups (CON + luteolin and CYP + luteolin) received daily gavage of luteolin (100 mg/kg, T2682, TCI, Japan) [16, 17, 18], while the CON and CYP groups were given a comparable volume of saline. On day five, the groups designated for CYP treatment (CYP and CYP + luteolin) were injected intraperitoneally with CYP, whereas saline was administered intraperitoneally to the CON and CON + luteolin groups. The experimental cycle concluded after 1 week with the harvesting of mouse bladders for further experimental analyses.

### 2.5 Hematoxylin and Eosin (HE) Staining and Histological Score and TUNEL Staining

Mouse bladder tissues underwent fixation in paraformaldehyde, followed by paraffin embedding. Histological evaluations were conducted using HE staining [19], with inflammation scores assigned based on established literature [20]. Apoptosis was assessed in bladder sections (*n* = 6) using TUNEL staining [21], which detects DNA cleavage with a Roche Diagnostics in‐situ cell death detection kit. The extent of apoptosis was quantitatively analyzed using ImageJ software to calculate the apoptosis index.

### 2.6 Enzyme‐Linked Immunosorbent Assay (ELISA)

Mouse bladder tissue (*n* = 6) homogenate was centrifuged, and the supernatant was extracted for ELISA detection. ELISA kit of malondialdehyde (MDA; K025‐M), total superoxide dismutase (SOD; K019‐M), glutathione (GSH; 0026c), interleukin 6 (IL‐6; M0044c), interleukin 1β (IL‐1β; M0037c), and interleukin 18 (IL‐18; M0730c) were purchased from Elabscience Biotechnology (Wuhan, China). All ELISA kits were used following the manufacturer's instructions, and the absorbance was measured at 450 nm with an ultraviolet microplate reader.

### 2.7 Western Blot Analysis

Western blot analysis was performed as previously described [22]. Forty micrograms of protein per sample (*n* = 6) were subjected to electrophoresis. Proteins were probed at 4°C overnight using antibodies against PI3K (1:1000, 67121‐1), AKT (1:800, 10176‐2), phosphorylated AKT (p‐AKT, 1:800, 28731‐1), p53 (1:1000, 21891‐Ig), phosphorylated p53 (p‐p53, 1:1000, 28961‐1), GAPDH (1:1000, 60004), Cleaved‐caspase‐3 (1:800, 66470), Cleaved‐Caspase‐8 (1:1000, 9496), and Bax (1:1000, 60267). Following three washes, the PVDF membranes were incubated with secondary antibodies (goat anti‐mouse, 1:3000, SA00001‐1 and goat anti‐rabbit, 1:2000, SA00001‐2). Protein bands were visualized and quantitatively analyzed for relative optical density using the ChemiDoc XRS + Image System.

### 2.8 Detection of Mouse Cystometry

Cystometry was performed based on previous research reports [23]. The experiment should be conducted gently to avoid animal panic, minimize animal pain, and ensure that the experimental animals are treated humanely and properly. The mice (*n* = 6) were anesthetized with an intraperitoneal injection of urethane (1 g/kg body weight) and catheterized via the urethra using a PE 10 catheter. The bladder was perfused at a rate of 1 mL/h, with continuous bladder pressure recording. Following a 30‐min stabilization period, urodynamic parameters such as micturition frequency (MF), intercontraction interval (ICI), and maximum bladder pressure (MBP) were measured.

### 2.9 Statistical Analysis

Statistical analyses were conducted using SPSS Statistics (version 22.0; SPSS Inc., USA). Data adhering to a normal distribution are presented as mean ± standard deviation (*M* ± SD). Group comparisons were made using t‐tests for normally distributed data. For categorical data, frequencies and percentages were used, and differences were assessed using the chi‐square test. For comparisons involving multiple groups, one‐way ANOVA or the Kruskal–Wallis test was applied based on the variance distribution, followed by post hoc analysis with the Student–Newman–Keuls test. The Bonferroni correction was employed for multiple comparisons adjustment. A *p*‐value of less than 0.05 was considered statistically significant, with significance levels marked as NS: not significant, *p < 0.05, **p < 0.01, ***p < 0.001.

## 3 Result

### 3.1 Screening of Active Ingredients of SM and Common Drug‐Disease Targets

**Figure 1:**
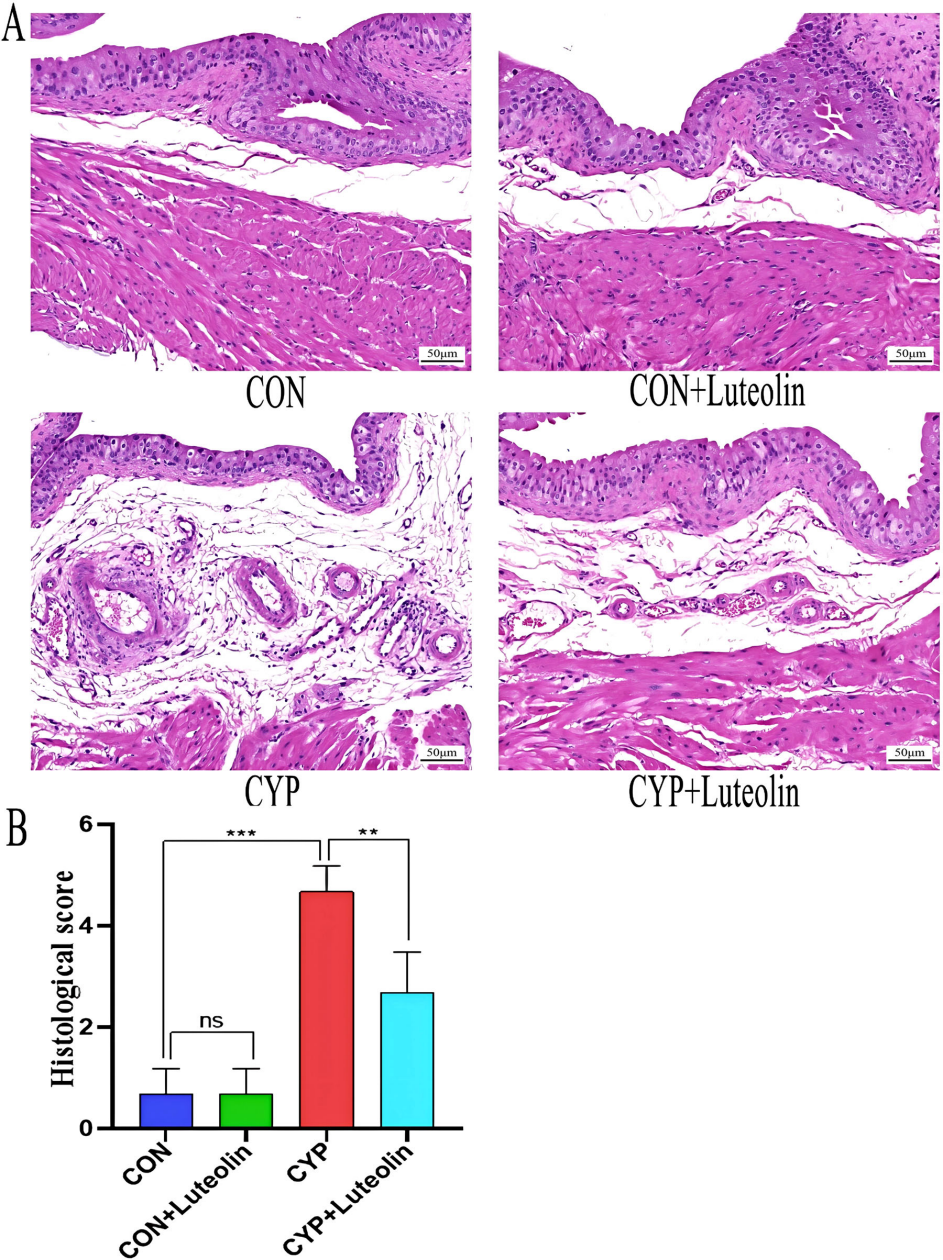
Luteolin improves bladder tissue damage in CYP‐induced cystitis. (A) Bladder microstructure (x200) from four groups of rats. In the CYP group, bladder epithelial injury, edema, bleeding, and inflammatory cell infiltration were observed. Bladder injury was significantly improved in CYP + LUT group. (B) Statistical chart (*n* = 6) shows the bladder histological score in the four groups. ***p < 0.001, **p < 0.01, *p < 0.05, ns represents no significant difference.

Under screening conditions of OB ≥ 30% and DL ≥ 0.18, 65 potential active ingredients and 758 potential drug targets of SM were obtained after deduplication. As shown in Table 1, the active ingredients of *S. miltiorrhiza* Bunge include salvianolic acids, tanshinones, tanshinones, and polysaccharide. Using “Bladder Pain Syndrome” and “Interstitial Cystitis” as keywords, the OMIM, Disgenet, and Genecards databases were searched, and 1462 IC/BPS targets were obtained after deduplication. A total of 148 common drug‐disease targets were obtained (Supporting Information S1: Figure 1A) by taking the intersection of drug targets and disease targets.

**Table 1:** Active components in *Salvia miltiorrhiza* bunge.

Mol ID	Molecule name	OB (%)	DL
MOL007064	Przewalskin b	110.32	0.44
MOL007132	(2R)‐3‐(3,4‐dihydroxyphenyl)‐2‐[(Z)‐3‐(3,4‐dihydroxyphenyl)acryloyl]oxy‐propionic acid	109.38	0.35
MOL007140	(Z)‐3‐[2‐[(E)‐2‐(3,4‐dihydroxyphenyl)vinyl]‐3,4‐dihydroxy‐phenyl]acrylic acid	88.54	0.26
MOL007150	(6S)‐6‐hydroxy‐1‐methyl‐6‐methylol‐8,9‐dihydro‐7H‐naphtho[8,7‐g]benzofuran‐10,11‐quinone	75.39	0.46
MOL007058	Formyltanshinone	73.44	0.42
MOL007120	Miltionone Ⅱ	71.03	0.44
MOL007105	Epidanshenspiroketallactone	68.27	0.31
MOL007155	(6S)‐6‐(hydroxymethyl)‐1,6‐dimethyl‐8,9‐dihydro‐7H‐naphtho[8,7‐g]benzofuran‐10,11‐dione	65.26	0.45
MOL007130	Prolithospermic acid	64.37	0.31
MOL007050	2‐(4‐hydroxy‐3‐methoxyphenyl)‐5‐(3‐hydroxypropyl)‐7‐methoxy‐3‐benzofurancarboxaldehyde	62.78	0.4
MOL007068	Przewaquinone B	62.24	0.41
MOL000569	Digallate	61.85	0.26
MOL007081	Danshenol B	57.95	0.56
MOL007082	Danshenol A	56.97	0.52
MOL007069	Przewaquinone c	55.74	0.4
MOL007108	Isocryptotanshi‐none	54.98	0.39
MOL007125	Neocryptotanshinone	52.49	0.32
MOL007079	Tanshinaldehyde	52.47	0.45
MOL007088	Cryptotanshinone	52.34	0.4
MOL007094	Danshenspiroketallactone	50.43	0.31
MOL007111	Isotanshinone II	49.92	0.4
MOL007154	Tanshinone iia	49.89	0.4
MOL007119	Miltionone Ⅰ	49.68	0.32
MOL007098	Deoxyneocryptotanshinone	49.4	0.29
MOL007048	(E)‐3‐[2‐(3,4‐dihydroxyphenyl)‐7‐hydroxy‐benzofuran‐4‐yl]acrylic acid	48.24	0.31
MOL007051	6‐o‐syringyl‐8‐o‐acetyl shanzhiside methyl ester	46.69	0.71
MOL007156	Tanshinone Ⅵ	45.64	0.3
MOL007141	Salvianolic acid g	45.56	0.61
MOL001942	Isoimperatorin	45.46	0.23
MOL007101	DihydrotanshinoneⅠ	45.04	0.36
MOL007115	Manool	45.04	0.2
MOL007123	Miltirone Ⅱ	44.95	0.24
MOL007045	3α‐hydroxytanshinoneⅡa	44.93	0.44
MOL001659	Poriferasterol	43.83	0.76
MOL002651	Dehydrotanshinone II A	43.76	0.4
MOL007077	Sclareol	43.67	0.21
MOL007142	Salvianolic acid j	43.38	0.72
MOL007152	Przewaquinone E	42.85	0.45
MOL007151	Tanshindiol B	42.67	0.45
MOL007070	(6S,7R)‐6,7‐dihydroxy‐1,6‐dimethyl‐8,9‐dihydro‐7H‐naphtho[8,7‐g]benzofuran‐10,11‐dione	41.31	0.45
MOL007041	2‐isopropyl‐8‐methylphenanthrene‐3,4‐dione	40.86	0.23
MOL007071	Przewaquinone f	40.31	0.46
MOL002776	Baicalin	40.12	0.75
MOL007118	Microstegiol	39.61	0.28
MOL006824	α‐amyrin	39.51	0.76
MOL007124	Neocryptotanshinone ii	39.46	0.23
MOL007093	Dan‐shexinkum d	38.88	0.55
MOL007122	Miltirone	38.76	0.25
MOL001601	1,2,5,6‐tetrahydrotanshinone	38.75	0.36
MOL007100	Dihydrotanshinlactone	38.68	0.32
MOL007063	Przewalskin a	37.11	0.65
MOL007061	Methylenetanshinquinone	37.07	0.36
MOL001771	Poriferast‐5‐en‐3beta‐ol	36.91	0.75
MOL007121	Miltipolone	36.56	0.37
MOL000006	Luteolin	36.16	0.25
MOL002222	Sugiol	36.11	0.28
MOL007107	C09092	36.07	0.25
MOL007127	1‐methyl‐8,9‐dihydro‐7H‐naphtho[5,6‐g]benzofuran‐6,10,11‐trione	34.72	0.37
MOL007149	NSC 122421	34.49	0.28
MOL007049	4‐methylenemiltirone	34.35	0.23
MOL007036	5,6‐dihydroxy‐7‐isopropyl‐1,1‐dimethyl‐2,3‐dihydrophenanthren‐4‐one	33.77	0.29
MOL007143	Salvilenone Ⅰ	32.43	0.23
MOL007059	3‐beta‐Hydroxymethyllenetanshiquinone	32.16	0.41
MOL007145	Salviolone	31.72	0.24
MOL007085	Salvilenone	30.38	0.38

### 3.2 Construction and Degree Value Analysis of SM Component‐Target‐IC/BPS Network

As shown in Supporting Information S1: Figure 1B, green represents the active components of SM and blue represents the common targets. The larger the circles and squares in the figure, the higher the degree value, and the more important the components and targets are. As shown in Table 2, based on the degree value of the active ingredients, the authors found that the luteolin in SM was the most active ingredient in the treatment of IC/BPS.

**Table 2:** Salvia‐ingredient‐target‐IC/BPS network diagram ranking of active components by degree (top 10).

Mol ID	Full name	Degree
MOL000006	Luteolin	51
MOL007050	2‐(4‐hydroxy‐3‐methoxyphenyl)‐5‐(3‐hydroxypropyl)‐7‐methoxy‐3‐benzofurancarboxaldehyde	36
MOL007069	Przewaquinone c	33
MOL007093	Dan‐shexinkum d	32
MOL007081	Danshenol B	31
MOL007082	Danshenol A	30
MOL007036	5,6‐dihydroxy‐7‐isopropyl‐1,1‐dimethyl‐2,3‐dihydrophenanthren‐4‐one	28
MOL007105	Epidanshenspiroketallactone	28
MOL007132	(2R)‐3‐(3,4‐dihydroxyphenyl)‐2‐[(Z)‐3‐(3,4‐dihydroxyphenyl)acryloyl]oxy‐propionic acid	27
MOL007077	Sclareol	26

### 3.3 Target Screening of Luteolin in the Treatment of IC/BPS

**Figure 2:**
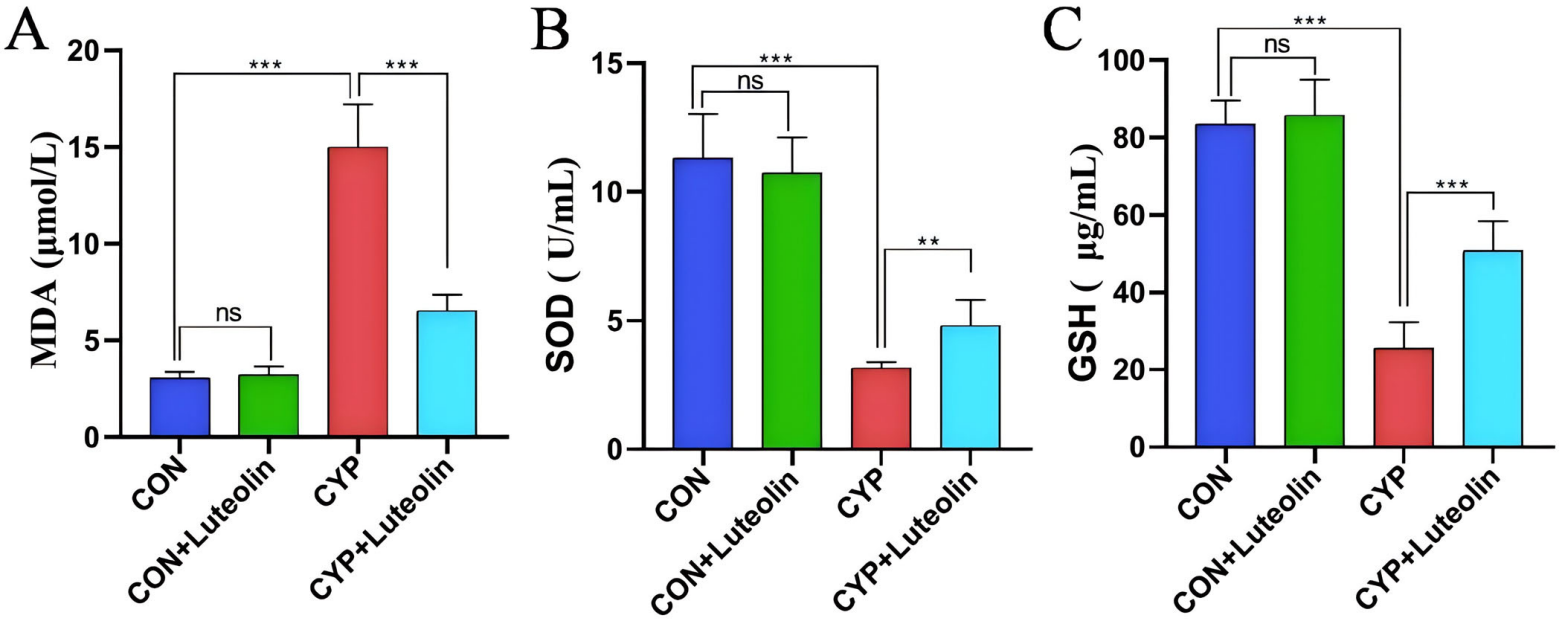
Luteolin reduces the expression of oxidative stress‐related indexes in CYP‐induced cystitis. The expressions of MDA (A), GSH (B), and total SOD (C) in the bladder tissue of the four groups were detected (*n* = 6). ***p < 0.001, **p < 0.01, *p < 0.05, ns represents no significant difference.

**Figure 3:**
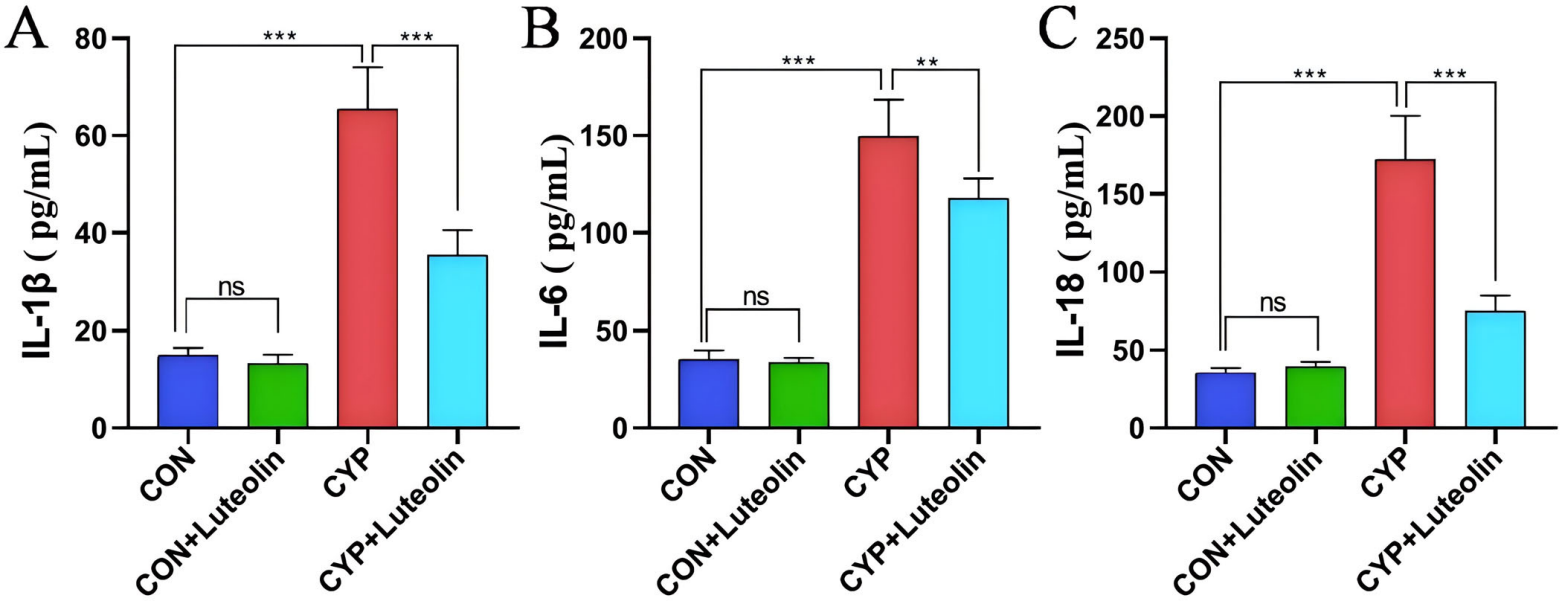
Luteolin reduces the expression of inflammatory factors in CYP‐induced cystitis. The expressions of IL‐1β (A), IL‐6 (B), and IL‐18 (C) in the bladder tissue of the four groups were detected (*n* = 6). ***p < 0.001, **p < 0.01, *p < 0.05, ns represents no significant difference.

Supporting Information S2: Figure 2A reveals the identification of 50 common drug‐disease targets. These targets were uploaded to Cytoscape software to generate a “disease‐target‐component” interaction network (Supporting Information S2: Figure 2B), a protein interaction network where the circle size and color intensity indicate higher degree values (Supporting Information S3: Figure 3A), and a PPI network diagram (Supporting Information S3: Figure 3B). Further analysis through PPI topological and clustering techniques, as detailed in Supporting Information S3: Figure 3C,D, pinpointed AKT1, VEGFA, TP53, EGFR, ERBB2, JUN, ESR1, CCND1, MMP‐9, and MDM2 as the core targets of luteolin in treating IC/BPS.

### 3.4 Luteolin GO Enrichment Analysis

**Figure 4:**
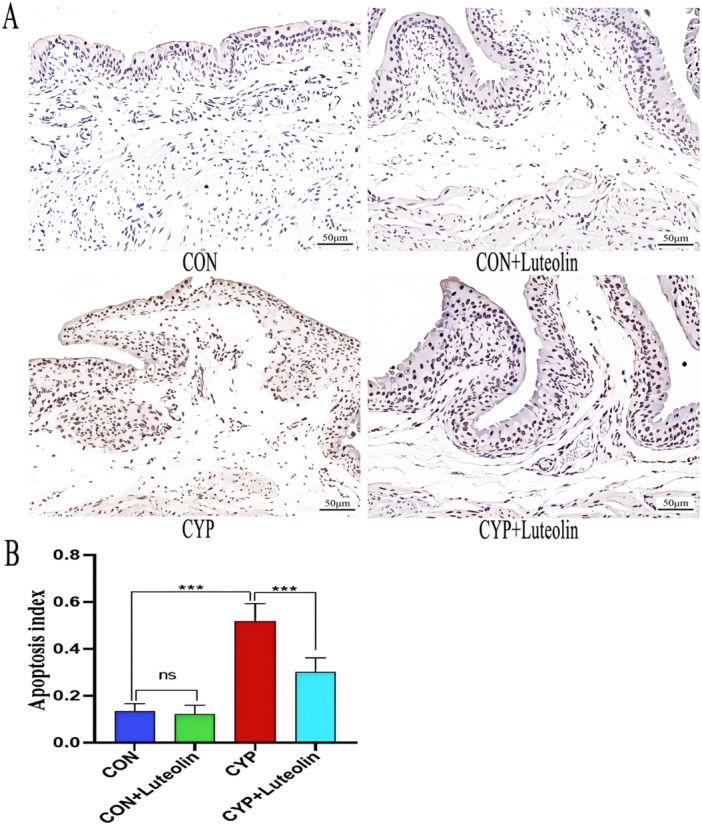
Luteolin reduces tissue apoptosis in CYP‐induced cystitis. (A) Four groups of bladder sections were stained with TUNEL, apoptotic cells were stained dark brown, and nonapoptotic cells were stained blue. (B) Statistics of apoptotic index from TUNEL staining in each group (*n* = 6). ***p < 0.001, **p < 0.01, *p < 0.05, ns represents no significant difference.

The GO enrichment analysis of luteolin identified that 50 intersecting genes were involved in 1285 BPs, 36 CCs, and 90 MFs. As displayed in Supporting Information S4: Figure 4, the top 20 GO enrichment results were visualized in a bar graph based on their combined scores. Prominent BPs enriched include cellular responses to chemical stress, responses to oxidative stress, and cellular responses to oxidative stress. Key MFs highlighted are phosphatase binding, RNA polymerase II‐specific DNA‐binding transcription factor binding, and DNA‐binding transcription factor binding. The principal CCs identified comprise the transferase complex, nuclear envelope, protein kinase complex, membrane raft, and membrane microdomains.

### 3.5 Analysis on Luteolin KEGG Target Pathway Annotation and Core Target Molecular Intersection

**Figure 5:**
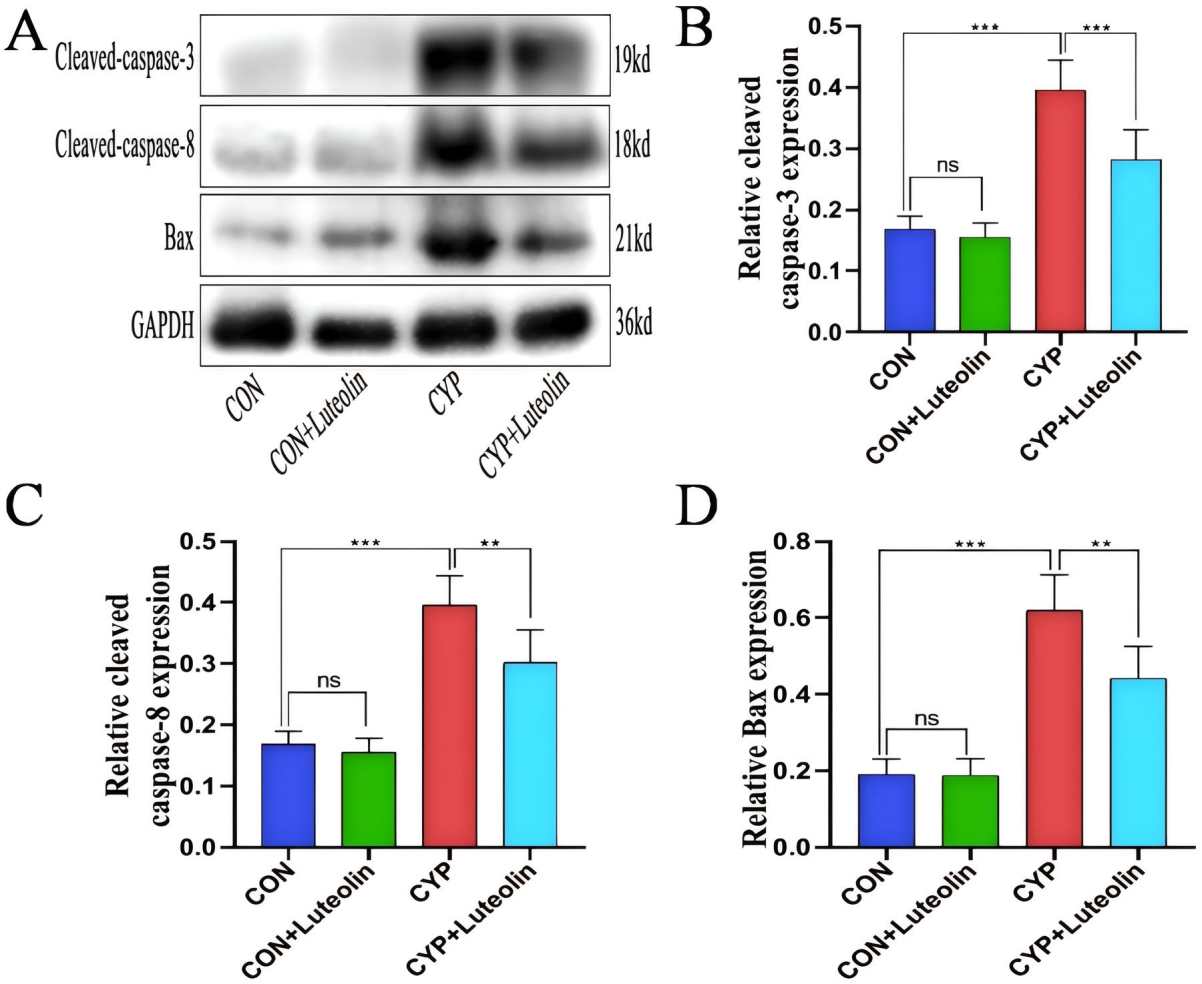
Luteolin reduces apoptosis‐related protein expression in CYP‐induced cystitis. (A) Western blot results show the expression levels of Bax, cleaved caspase‐3, and cleaved caspase‐8 in the four groups. (B–D) The statistical chart shows the statistical analysis of the western blot results in the four groups (*n* = 6). ***p < 0.001, **p < 0.01, *p < 0.05, ns represents no significant difference.

According to the Luteolin KEGG analysis, 50 intersecting genes were enriched in 129 KEGG pathways. As shown in Supporting Information S5: Figure 5A, the first 20 KEGG enrichment results were obtained based on the *p*‐values displayed in the bar graph. The authors constructed a “component‐target‐pathway” network map, integrating 20 pathways with their related targets before enrichment (Supporting Information S5: Figure 5B), where the size of the blue circles indicates the higher degree value of the target molecules. This network map revealed that genes in the KEGG pathways, particularly those associated with tumor‐related pathways, PI3K‐Akt signaling, and HIF‐1 signaling, were notably abundant. Subsequent analysis focused on intersecting the target molecules that ranked in the top 10 in both the luteolin KEGG target pathway and the luteolin PPI topology analysis. This revealed TP53, AKT1, CCND1, EGFR, and ERBB2 as critical core targets of luteolin in the treatment of IC/BPS, as depicted in Supporting Information S5: Figure 5C. Based on the literature reports combined with the results of the network pharmacology in this study, the authors found that the PI3K‐Akt and TP53 signaling‐related pathways were important core pathways for luteolin to play a role in the treatment of IC/BPS [16, 17, 18, 24, 25, 26].

### 3.6 Improvement of Bladder Tissue Damage and Reduction in Expression of Inflammatory Factors and Oxidative Stress‐Related Indicators in CYP‐Induced Cystitis by Luteolin

Figure 1A demonstrates that the bladder epithelium of mice in both the CON and CON + luteolin groups was intact, showing no significant pathological changes. In contrast, the bladders of mice in the CYP group exhibited severe hemorrhage, edema, epithelial damage, and extensive inflammatory cell infiltration. Notably, the CYP + luteolin group showed marked improvements in bladder epithelial damage, hemorrhage, edema, and inflammatory infiltration, with a significant reduction in the histological score compared to the CYP group (0.52 ± 0.08 vs. 0.30 ± 0.06, *p* < 0.001). As detailed in Figure 2A–C, bladder tissues from the CYP group displayed significantly reduced levels of total SOD and GSH, and elevated levels of MDA (all *p* < 0.001) compared to the CON and CON + luteolin groups. However, treatment with luteolin in the CYP + luteolin group resulted in significantly lower MDA levels (15.01 ± 2.23 vs. 6.54 ± 0.83, *p* < 0.001) and higher levels of GSH (25.52 ± 6.75 vs. 50.75 ± 7.74, *p* < 0.001) and total SOD (3.16 ± 0.24 vs. 4.81 ± 0.99, *p* = 0.002) compared to the CYP group alone. Further, as illustrated in Figure 3A–C, inflammatory cytokines such as IL‐1β, IL‐6, and IL‐18 were significantly elevated in the CYP group compared to the CON and CON + luteolin groups (all *p* < 0.001). Conversely, the CYP + luteolin group showed significantly decreased levels of IL‐1β (65.41 ± 8.71 vs. 35.49 ± 5.12, *p* < 0.001), IL‐6 (149.58 ± 18.95 vs. 117.75 ± 10.27, *p* = 0.004), and IL‐18 (74.91 ± 9.91 vs. 35.58 ± 3.16, *p* < 0.001) relative to the CYP group. These findings underscore the antioxidant and anti‐inflammatory properties of luteolin in mitigating bladder tissue damage.

### 3.7 Luteolin Reduces Tissue Apoptosis in CYP‐Induced Cystitis

As shown in Figure 4, compared with the CON and CON + luteolin groups, the CYP group had a significantly increased bladder apoptosis index (*p* < 0.001). Meanwhile, as shown in Figure 5, the expression of apoptosis‐related proteins cleaved caspase‐3 (*p* < 0.001), Bax (*p* < 0.001), and cleaved caspase‐8 (*p* < 0.001) was significantly upregulated. However, compared with the CYP group, the CYP + luteolin group had a significantly decreased bladder apoptosis index (Figure 4) (0.52 ± 0.08 vs. 0.31 ± 0.06, *p* < 0.001), with the apoptosis‐related protein Bax (0.62 ± 0.09 vs. 0.44 ± 0.08, *p* < 0.001), cleaved caspase‐3 (0.44 ± 0.05 vs. 0.31 ± 0.05, *p* < 0.001), and cleaved caspase‐8 (0.40 ± 0.05 vs. 0.30 ± 0.05, *p* < 0.001) (Figure 5). These results suggest that luteolin has antiapoptotic properties.

### 3.8 Luteolin Inhibits the Activation of PI3K/AKT and p53 Signal Pathway in CYP‐Induced Cystitis

**Figure 6:**
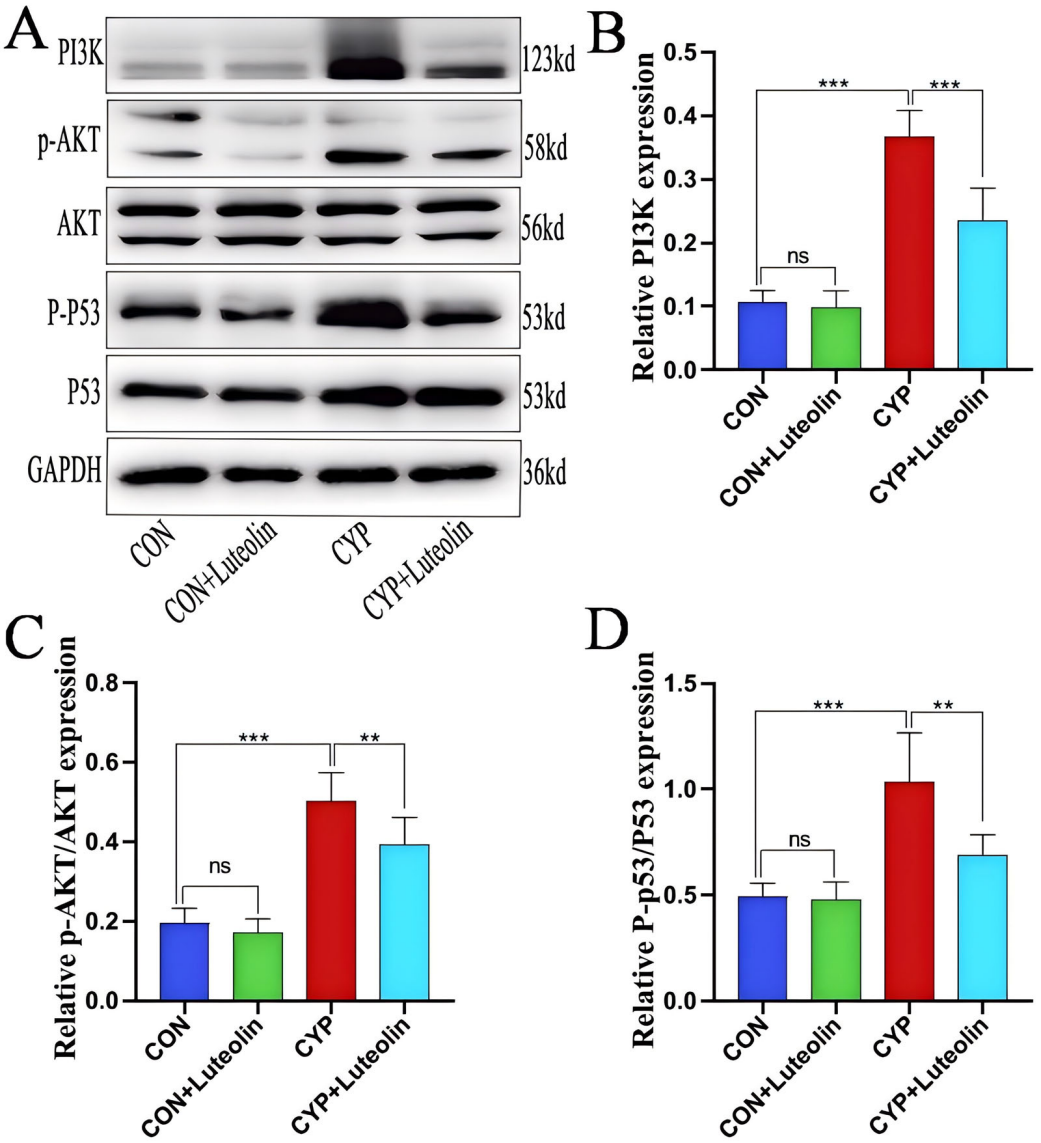
Luteolin inhibits the activation of PI3K/AKT and P53 signal pathway in CYP‐induced cystitis. (A) Western blot results show the expression levels of p53, p‐p53, AKT, p‐AKT, and PI3K in the four groups. (B–D) The statistical chart shows the statistical analysis of the western blot results in the four groups (*n* = 6). ***p < 0.001, **p < 0.01, *p < 0.05, ns represents no significant difference.

The regulatory effects of PI3K‐Akt and p53 signaling axes on inflammation, oxidative stress, and apoptosis have been widely reported [16, 17, 18, 24, 25, 26, 27]. In this study, the authors found that the core pathways of luteolin in the treatment of IC/BPS were PI3K/AKT and p53 through network pharmacology research. Therefore, we examined changes in the expression of PI3K/AKT and p53 in the CYP‐induced cystitis after luteolin intervention. The expression of p‐p53 (*p* < 0.001), PI3K (*p* < 0.001), and p‐Akt (*p* < 0.001) was significantly increased in the CYP group compared with those in the CON and CON + luteolin groups (Figure 6). However, compared with the CYP group, the CON and CON + luteolin groups had significantly decreased expressions of p‐p53 (1.03 ± 0.23 vs. 0.67 ± 0.01, *p* = 0.020), PI3K (0.37 ± 0.04 vs. 0.24 ± 0.05, *p* < 0.001), and p‐Akt (0.50 ± 0.07 vs. 0.39 ± 0.07, *p* = 0.009). These results suggest that luteolin can inhibit the signal transduction axis of PI3K‐Akt and p53.

### 3.9 Improvement of Bladder Function in CYP‐Induced Cystitis by Luteolin

**Figure 7:**
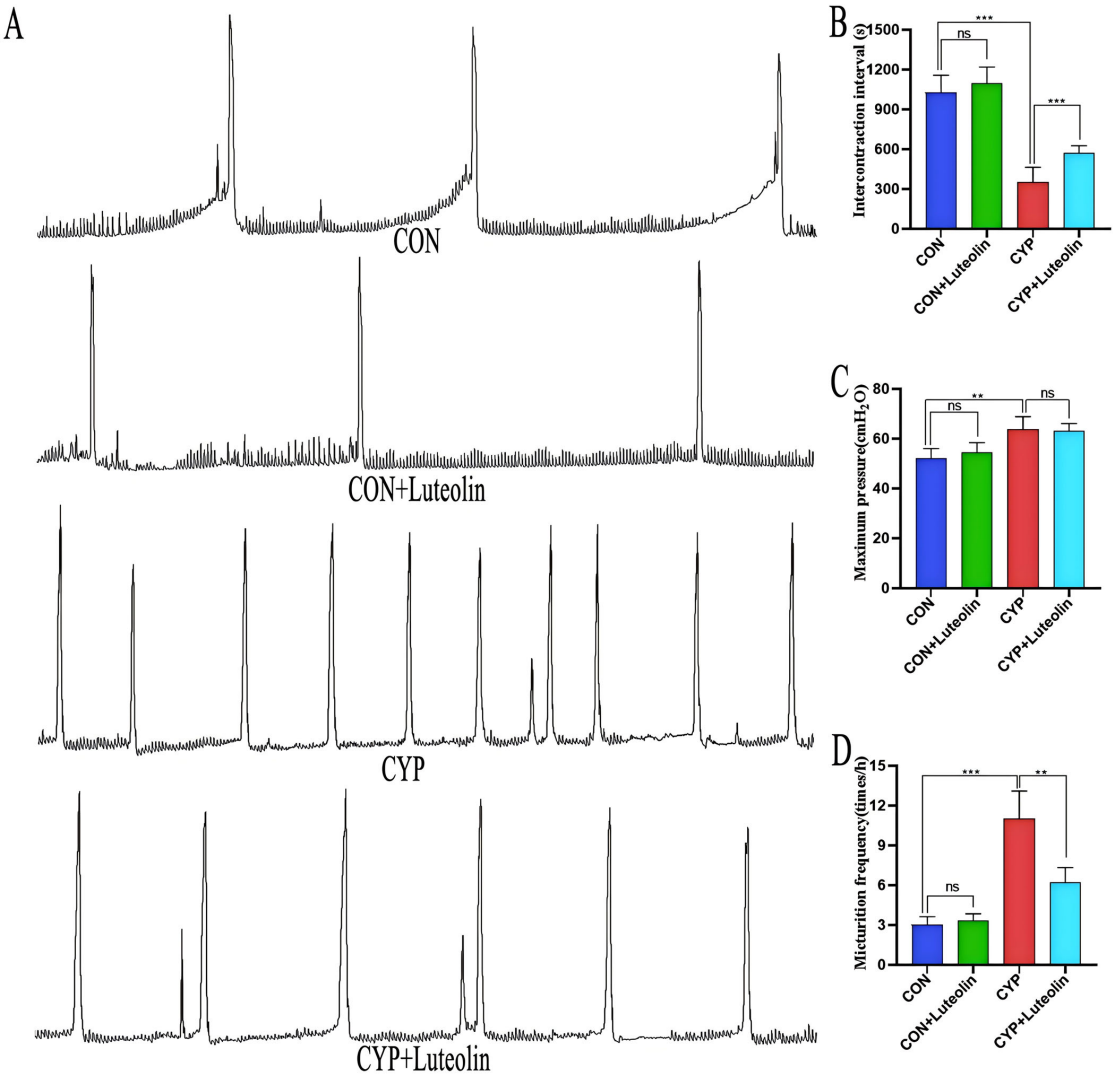
Luteolin improves bladder function in CYP‐induced cystitis. (A) Cystometry shows the changes of bladder function in four groups. (B–D) Statistical chart of the four groups micturition frequency, maximum pressure, and intercontraction interval (*n* = 6). ***p < 0.001, **p < 0.01, *p < 0.05, ns represents no significant difference.

Compared with the CON and CON + Luteolin groups (Figure 7), the CYP group had significantly increased bladder MBP (*p* < 0.014) and MF (*p* < 0.001), with ICI significantly shortened (*p* < 0.001), suggesting that the contractile function of the mouse bladders was significantly impaired. However, compared with the CYP group, the CYP + Luteolin group had a significantly decreased MF (11.0 ± 2.097 vs. 6.166 ± 1.169, *p* = 0.004), with the ICI significantly prolonged (348.98 ± 115.57 vs. 569.09 ± 57.16, *p* < 0.001), suggesting that the mouse bladder contractile function was significantly improved. These results suggest that luteolin improves bladder function in CYP‐induced cystitis.

## 4 Discussion

The pathogenesis of IC/BPS is a complex process. Current theories suggest that infection, epithelial permeability changes, mast cell activation, autoimmune responses, neuroinflammation, and other factors are involved in the pathophysiological process of IC/BPS [28, 29, 30]. However, existing evidence suggests that disorders of inflammation and immune regulation lead to persistent progression and failure to heal IC/BPS [28, 29, 30]. Exploring natural active ingredients that possess anti‐inflammatory and immunomodulatory properties represents a novel approach to treating IC/BPS. SM, a valued traditional Chinese medicine, has been extensively utilized in managing various conditions, including cardiovascular diseases, diabetes, arthritis, asthma, and other inflammatory disorders [8, 9, 10, 11]. Consequently, the authors employed network pharmacology to systematically analyze and identify the active components, key targets, and crucial signaling pathways of SM in the treatment of IC/BPS. In this study, we found that SM played a role in the treatment of IC/PBS through multiple components, multiple pathways, and multiple targets, including 65 potential active components (salvianolic acids, tanshinones, tanshinones, luteolin, etc.) and 758 potential drug targets. In a further active ingredient ranking analysis, the authors found that luteolin in SM was the most active ingredient in the treatment of IC/PBS.

Luteolin is a natural, weakly acidic tetrahydroxyflavonoid compound with strong antioxidant, anti‐inflammatory, antiapoptotic, and immunomodulatory activities [31, 32]. It has shown good therapeutic effects in gout, asthma, psoriasis, diabetes, cardiovascular and other diseases [31, 32]. In this study, we performed network pharmacology analysis on luteolin and found 50 common drug‐disease targets, among which AKT1, VEGFA, TP53, EGFR, ERBB2, JUN, ESR1, CCND1, MMP‐9, and MDM2 were the core targets for IC/BPS treatment. At the same time, the GO enrichment analysis of luteolin in this paper confirmed that luteolin could play physiological roles in cells and nucleus, and it played a physiological role mainly by regulating BPs such as cellular stress response, inflammatory and immune response, protein complex formation, DNA and RNA transcription factor binding. Finally, KEGG analysis of luteolin and intersection analysis of core target genes confirmed that TP53, AKT1, CCND1, EGFR, and ERBB2 were the core and important targets of luteolin in IC/BPS treatment. We reviewed the literature and combined the findings of network pharmacology [16, 17, 18, 24, 25, 26]. In this study, the authors speculated that the PI3K‐Akt and p53 signaling pathways are important pathways of luteolin in the treatment of IC/BPS. To explore the potential therapeutic efficacy of luteolin in IC/BPS, we conducted an intervention study on CYP‐induced cystitis using luteolin.

CYP is a commonly used chemotherapeutic drug, and its metabolite acrolein can induce bladder inflammation. CYP‐induced cystitis is widely used as an animal model to study the pathophysiology of IC/BPS [15, 22, 23, 33, 34, 35, 36]. CYP‐induced cystitis is often characterized by decreased bladder compliance, increased urination frequency, bladder epithelial injury, apoptosis of bladder epithelial cells and inflammatory cells, submucosal hemorrhage, inflammatory cell infiltration, significantly upregulated inflammatory factors (especially important inflammatory factors of IL‐1β, IL‐6 and IL‐18), apoptosis‐related proteins and oxidative stress‐related substances in bladder tissue [15, 22, 23, 33, 34, 35, 36]. In this study, CYP‐treated mice also showed a significant increase in MF and MBP, significant ICI shortening, bladder epithelial injury, inflammatory cell infiltration, bladder apoptosis index, apoptosis‐related proteins (Bax, cleaved caspase‐3, and cleaved caspase‐8), a significant increase in inflammatory factors (IL‐1β, IL‐6, and IL‐18), oxidative stress‐related substances (MDA), and a significant decrease in antioxidant stress‐related substances (GSH and total SOD). Luteolin is the main flavonoid in SM and can block the expression of oxidative stressors, inflammatory factors and apoptosis‐related proteins, showing strong activities of antioxidation, anti‐inflammation, and antiapoptosis [31, 32, 37, 38, 39]. Previous studies have shown that blocking inflammation and oxidative stress can significantly improve tissue damage and bladder functions in CYP‐induced cystitis [15, 22, 23, 33, 34, 35, 36]. In this study, luteolin‐treated mice showed a significant decrease in MF, bladder tissue score, apoptosis index, inflammatory factors, apoptosis‐related proteins, oxidative stress‐related substances (MDA), and a significant increase in ICI and antioxidant stress‐related substances (GSH and total SOD). These results suggest that luteolin can significantly block the inflammatory and oxidative stress responses of CYP‐induced cystitis and improve bladder tissue damage and function.

PI3K‐Akt and p53 are among the main signaling pathways that regulate oxidative stress, inflammatory response, and apoptosis in the body [40, 41, 42]. Current studies suggest that most flavonoids exert their biological activities of anti‐inflammation, anti‐oxidation, and antiapoptosis mainly through PI3K‐Akt and p53 [41, 42]. In pneumonia, asthma, hepatitis, and kidney injury diseases, luteolin can exert its biological activities by blocking the PI3K‐Akt and p53 signaling pathways [16, 17, 18, 24, 25, 26, 43, 44]. In this study, based on network pharmacology, the authors found that the PI3K‐Akt and p53 signaling pathways were the core pathways in which luteolin plays a role in the treatment of IC/BPS. Accordingly, we examined the PI3K‐Akt and p53 signaling pathways in CYP‐induced cystitis. The present study found that the PI3K‐Akt and p53 signaling pathways were activated in a CYP‐induced mouse cystitis model. However, using luteolin to interfere with CYP‐induced cystitis, the authors found that the protein expression of p‐Akt and p‐p53 was significantly reduced, and activation of the PI3K‐Akt and p53 signaling pathways was significantly blocked. Based on the findings from network pharmacology and animal experiments, it is proposed that luteolin could exert anti‐inflammatory, antioxidative, and antiapoptotic effects. These actions are likely mediated by the inhibition of the PI3K‐Akt and p53 signaling pathways, which contribute to improved bladder function.

In summary, the study indicates that SM may effectively treat IC/BPS through its multiple components, pathways, and targets. Luteolin, a key active component of SM, appears to inhibit the activation of the PI3K‐Akt and p53 pathways, thereby mitigating oxidative stress, inflammation, and apoptosis. This leads to reduced tissue inflammation and damage in the bladder, ultimately enhancing bladder tissue repair and contractile function.

## Author Contributions

**Liang Wang:** conceptualization, investigation, methodology, supervision, validation, writing – original draft, writing – review and editing. **Bei Yu:** conceptualization, data curation, formal analysis, funding acquisition, methodology, resources, software, validation, writing – original draft, writing – review and editing. **YaRong Wang:** conceptualization, data curation, formal analysis, methodology, supervision, validation, writing – original draft, writing – review and editing. **Xi Qu:** conceptualization, data curation, formal analysis, methodology, validation, writing – original draft, writing – review and editing. **Wei Tang:** conceptualization, data curation, methodology, supervision, validation, visualization, writing – review and editing.

## Ethics Statement

The study complied with the Animal Welfare Guidelines and Declaration of Helsinki and was authorized by the Laboratory Animal Welfare and Ethics Committee of the First Affiliated Hospital of Chongqing Medical University (CYFY‐20230109). This study adheres to internationally accepted standards for animal research, following the 3Rs principle. The ARRIVE guidelines were employed for reporting experiments involving live animals, promoting ethical research practices.

## Conflicts of Interest

The authors declare no conflicts of interest.

## Supporting information


**Supporting Figure 1 Screening of active components of** ***Salvia miltiorrhiza*** **Bunge and analysis of drug‐disease targets**. (A) Drug‐disease target map of *Salvia miltiorrhiza* Bunge and IC/BPS. (B) Compositions‐Targets network of *Salvia miltiorrhiza* Bunge. The blue circle represents the target, and the green square (DS represents *Salvia miltiorrhiza* Bunge) represents the active component of *Salvia miltiorrhiza* Bunge, the larger the circle and square, the higher the degree value.

**Supporting Figure 2 Analysis of common target of luteolin drug‐disease.** A is a Venn diagram of common drug‐disease targets. B is a network diagram of drug‐target‐disease interactions.

**Supporting Figure 3 Drug‐disease common target protein interaction analysis**. A is the core target protein protein interaction network diagram. B is the core target protein PPI network diagram. C and D are core target topology analysis and protein interaction analysis, respectively (Top 20).

**Supporting Figure 4 GO enrichment analysis of luteolin (Top 20)**. A is the biological process, B is the molecular function and C is the cellular component.

**Supporting Figure 5 KEGG enrichment analysis of luteolin (Top 20).** A is KEGG pathway enrichment bubble map (top 20). B is Interaction network diagram of Composition‐Target‐Pathway (blue the target; red the KEGG pathway, the greater the blue and red, the greater the degree value).

## Data Availability

Data used to support the findings of this study are available from the corresponding author upon request.
