# Supplementary material for: Active Compounds, Targets, and Mechanisms of Salvia miltiorrhiza Bunge in Treating Interstitial Cystitis/Bladder Pain Syndrome
Source: Immun Inflamm Dis. 2025 Apr 14;13(4):e70173. doi: 10.1002/iid3.70173 (PMC11995424; doi:10.1002/iid3.70173)
Supplement: Supplementary file 1 — Supporting Figure 1 Screening of active components of Salvia miltiorrhiza Bunge and analysis of drug‐disease targets. (A) Drug‐disease target map of Salvia miltiorrhiza Bunge and IC/BPS. (B) Compositions‐Targets network of Salvia miltiorrhiza Bunge. The blue circle represents the target, and the green square (DS represents Salvia miltiorrhiza Bunge) represents the active component of Salvia miltiorrhiza Bunge, the larger the circle and square, the higher the degree value. [file IID3-13-e70173-s005.pdf]

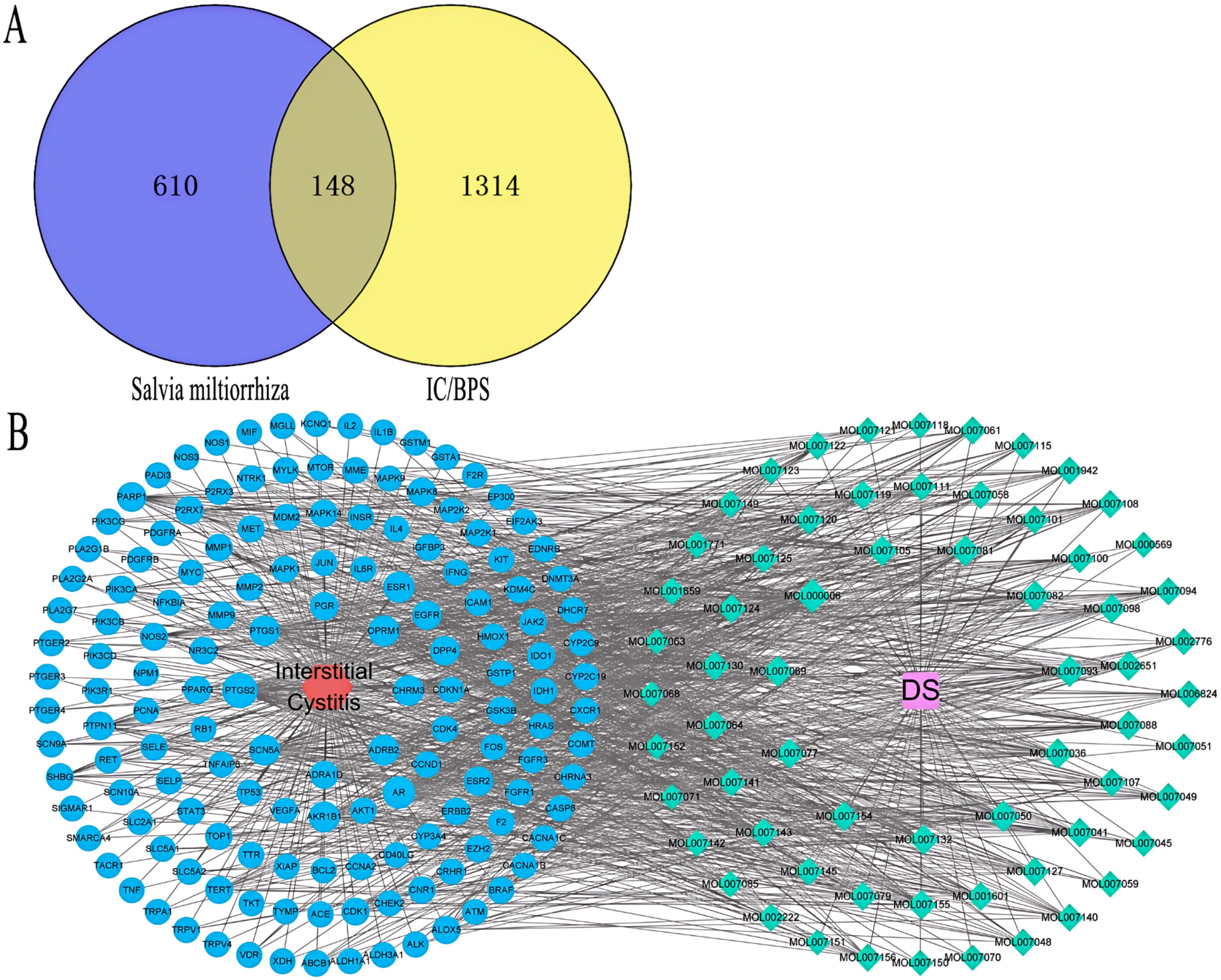
A
610
148
1314
Salvia miltiorrhiza
IC/BPS
B
Interstitial
Cystitis
DS
