# Supplementary figures and images for: Active Compounds, Targets, and Mechanisms of Salvia miltiorrhiza Bunge in Treating Interstitial Cystitis/Bladder Pain Syndrome
Source: Immun Inflamm Dis. 2025 Apr 14;13(4):e70173. doi: 10.1002/iid3.70173 (PMC11995424; doi:10.1002/iid3.70173)

A

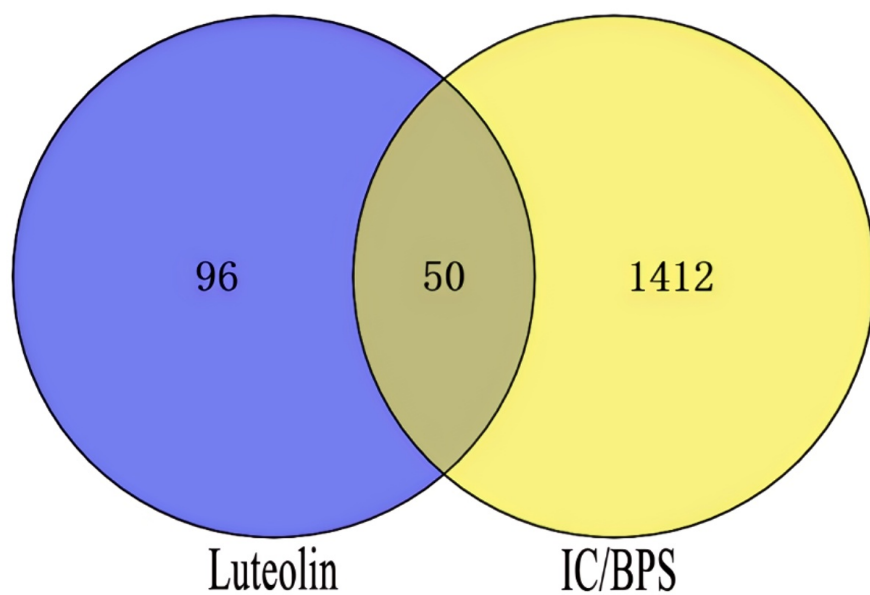

B

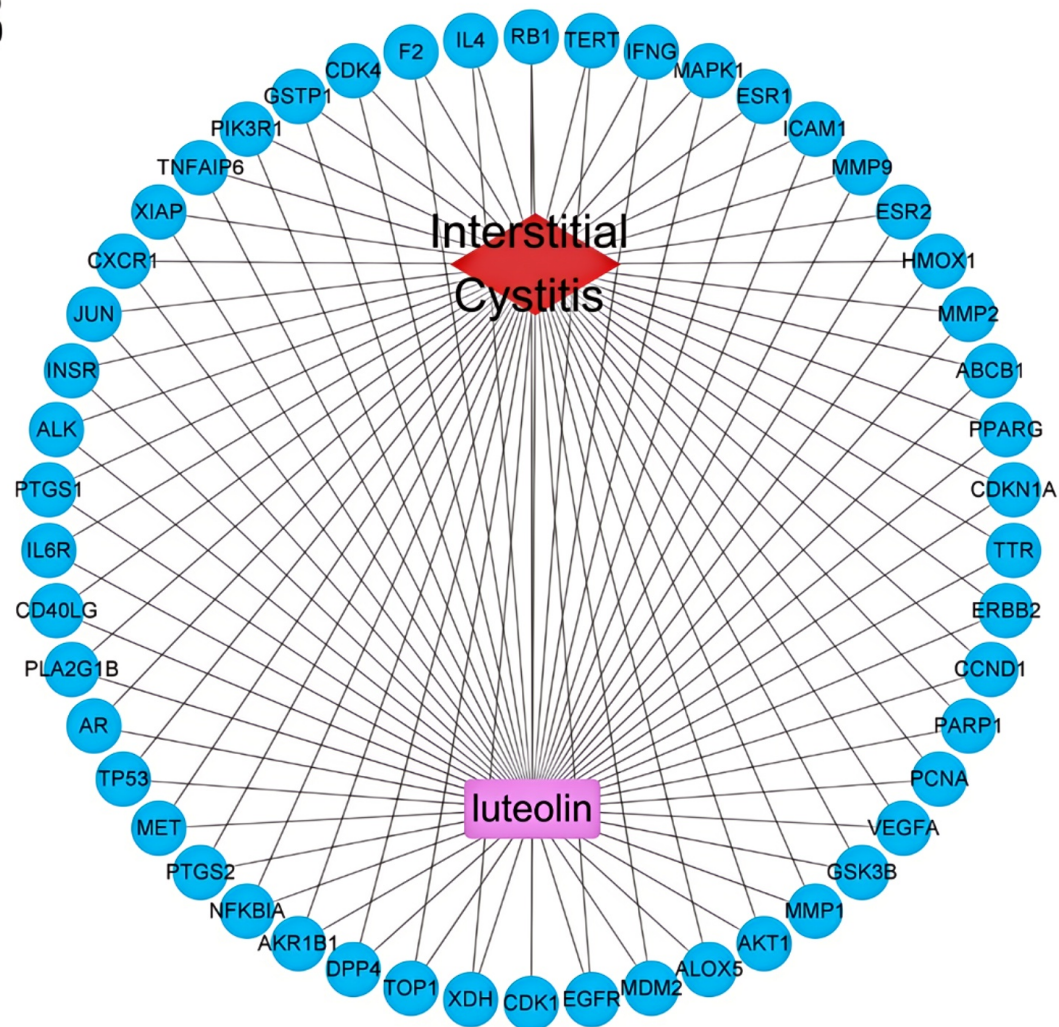

Supplement: Supplementary file 2 — Supporting Figure 2 Analysis of common target of luteolin drug‐disease. A is a Venn diagram of common drug‐disease targets. B is a network diagram of drug‐target‐disease interactions. [file IID3-13-e70173-s004.pdf]

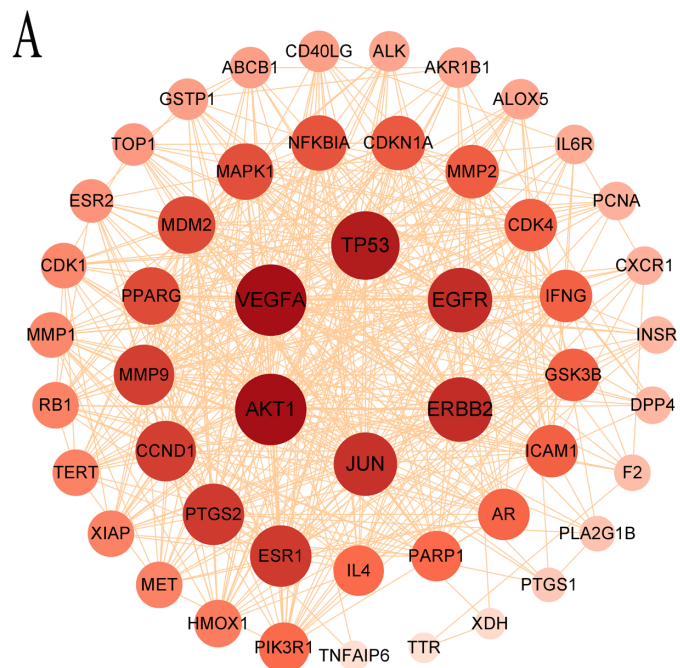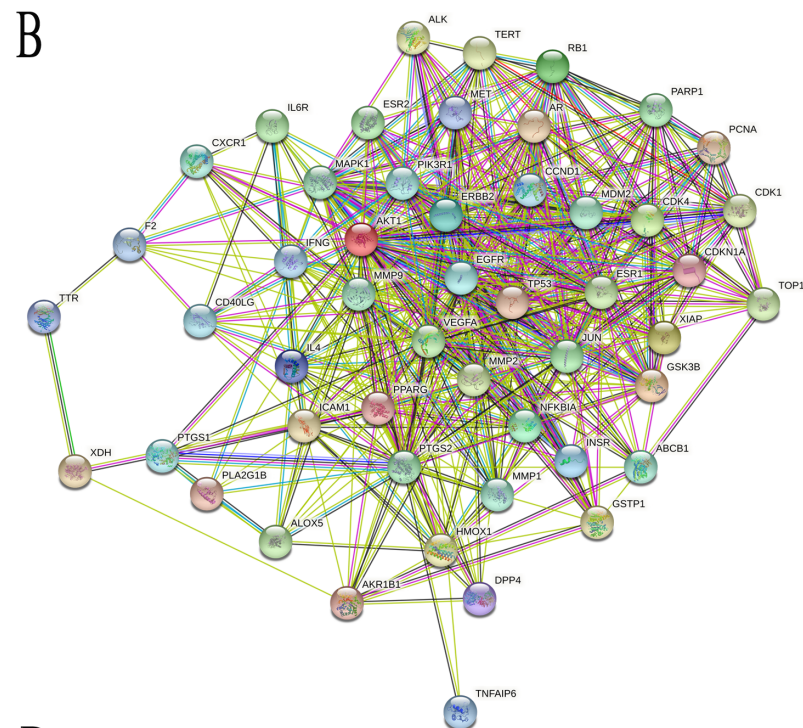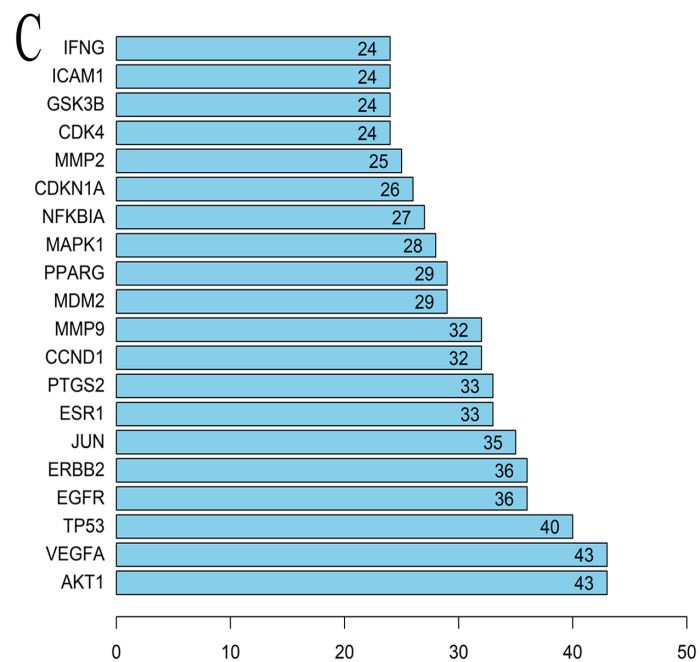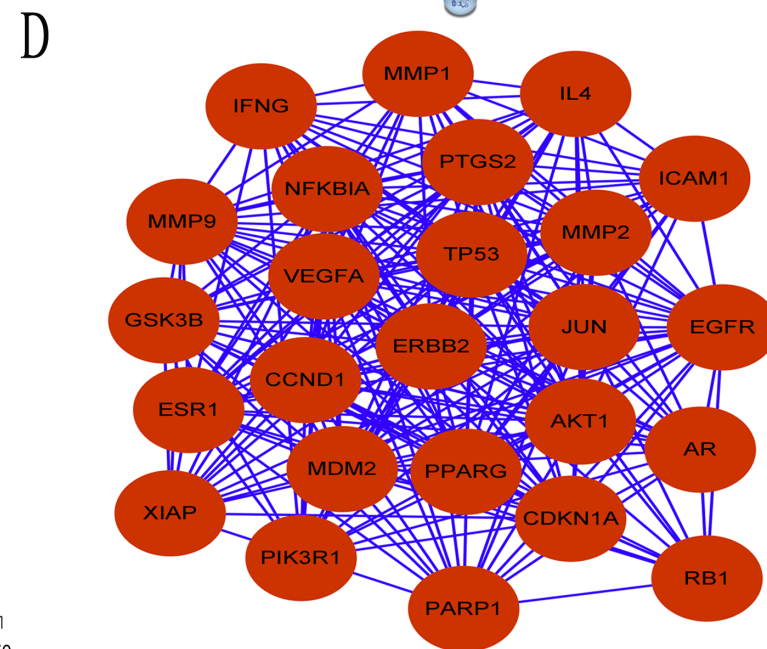

Supplement: Supplementary file 3 — Supporting Figure 3 Drug‐disease common target protein interaction analysis. A is the core target protein protein interaction network diagram. B is the core target protein PPI network diagram. C and D are core target topology analysis and protein interaction analysis, respectively (Top 20). [file IID3-13-e70173-s003.pdf]

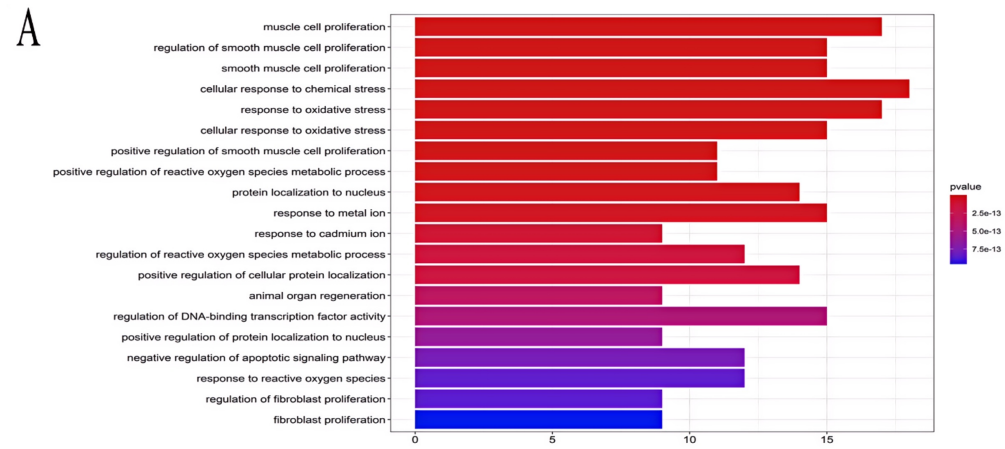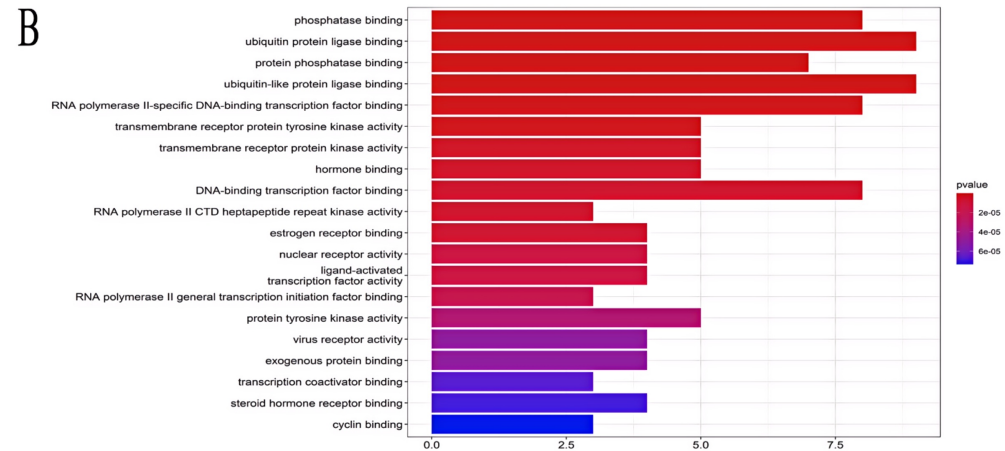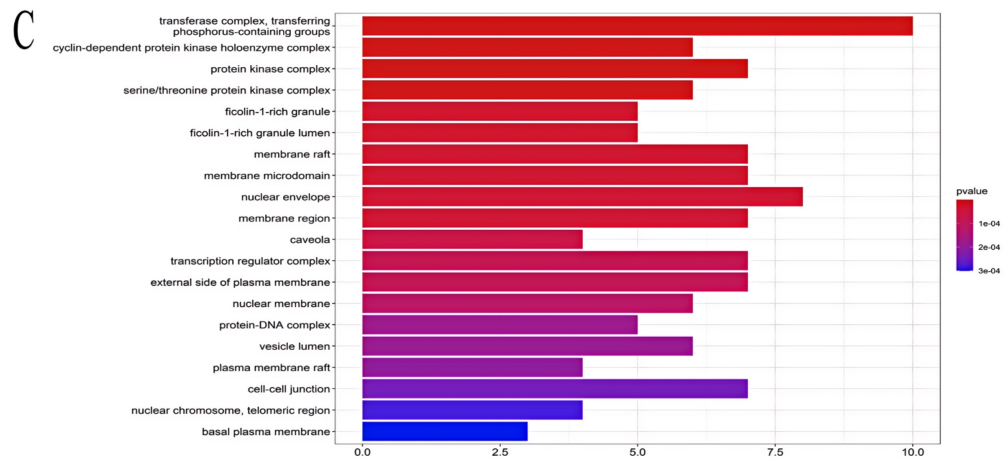

Supplement: Supplementary file 4 — Supporting Figure 4 GO enrichment analysis of luteolin (Top 20). A is the biological process, B is the molecular function and C is the cellular component. [file IID3-13-e70173-s001.pdf]

A

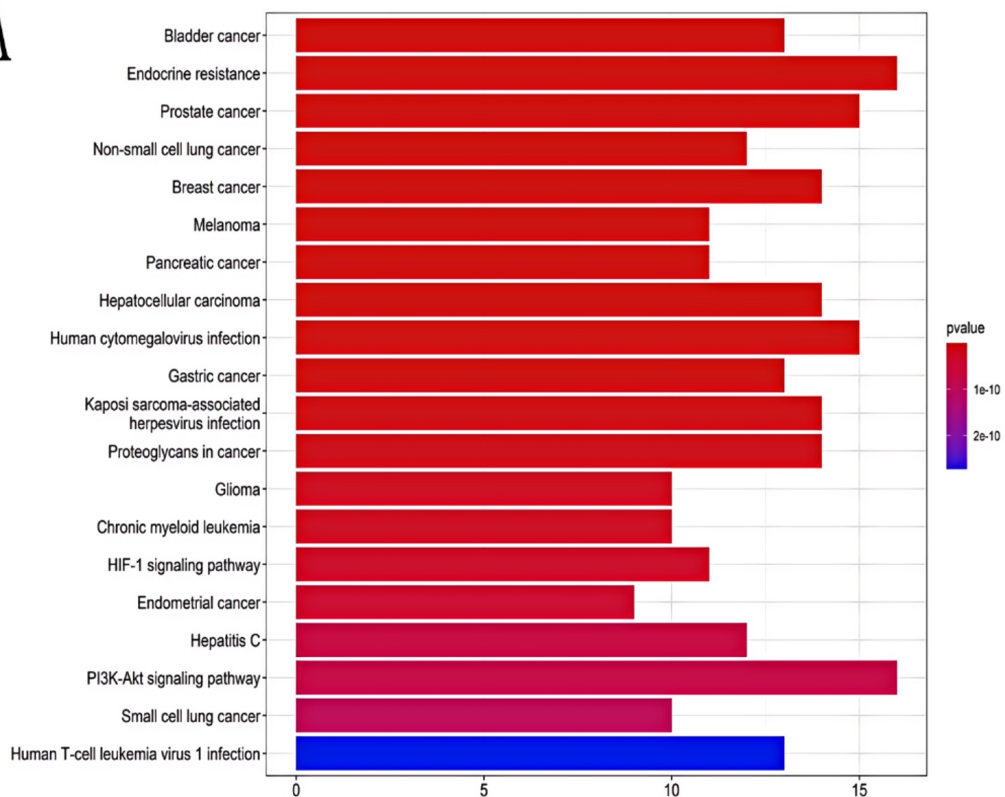

B

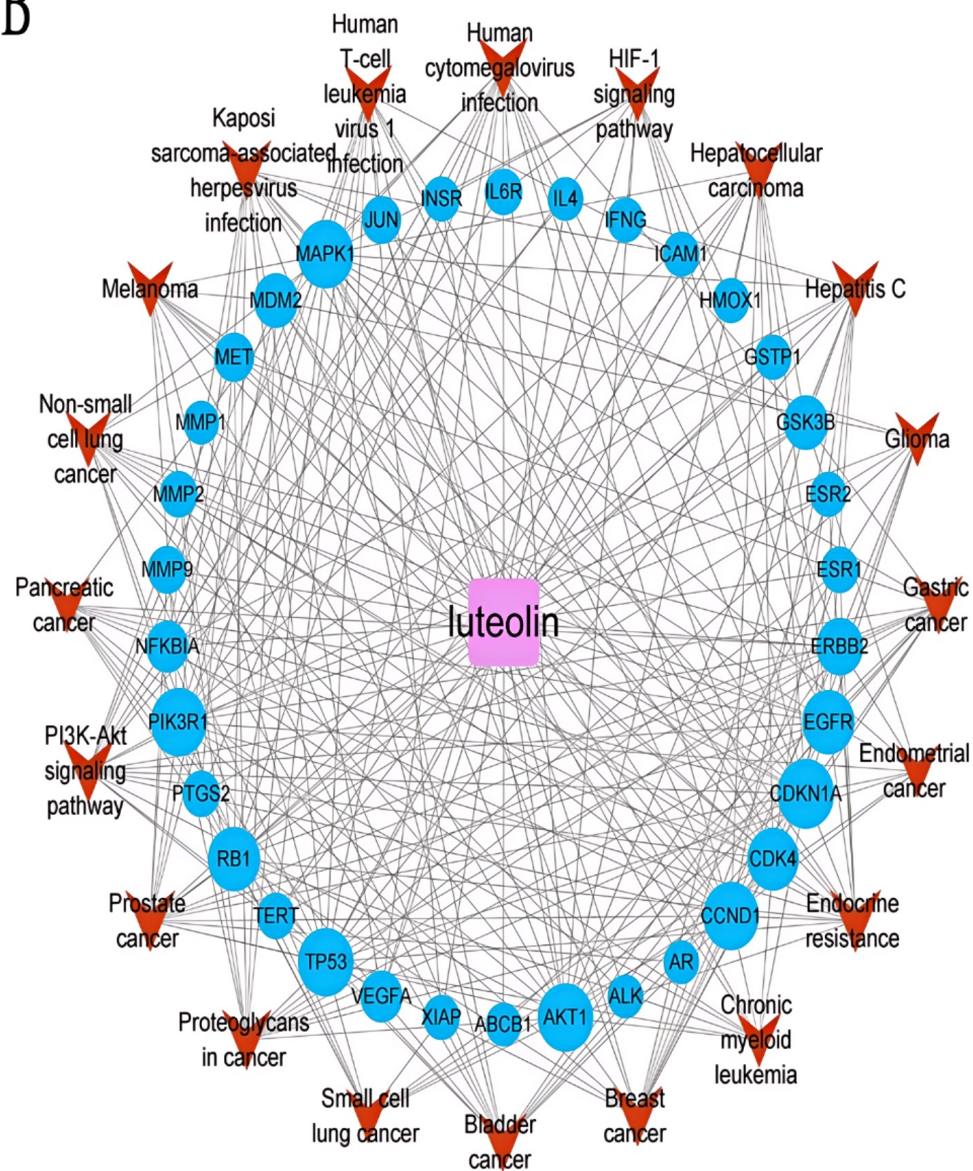

C

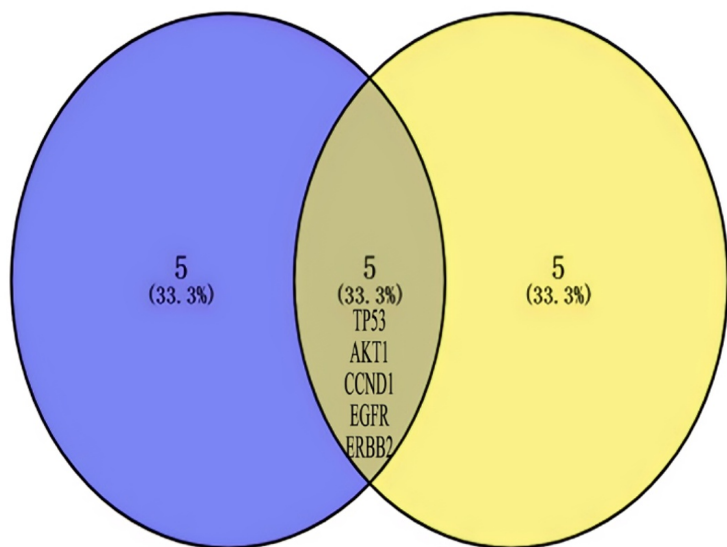

Supplement: Supplementary file 5 — Supporting Figure 5 KEGG enrichment analysis of luteolin (Top 20). A is KEGG pathway enrichment bubble map (top 20). B is Interaction network diagram of Composition‐Target‐Pathway (blue the target; red the KEGG pathway, the greater the blue and red, the greater the degree value). [file IID3-13-e70173-s002.pdf]
